# Cerebellar Cortex 4–12 Hz Oscillations and Unit Phase Relation in the Awake Rat

**DOI:** 10.3389/fnsys.2020.475948

**Published:** 2020-11-10

**Authors:** Maxime Lévesque, HongYing Gao, Carla Southward, J. M. Pierre Langlois, Clément Léna, Richard Courtemanche

**Affiliations:** ^1^Department of Neurology and Neurosurgery, Montreal Neurological Institute, McGill University, Montréal, QC, Canada; ^2^Institut de Biologie, CNRS UMR 8197–U 1024, École Normale Supérieure, Paris, France; ^3^Department of Health, Kinesiology and Applied Physiology, Center for Studies in Behavioral Neurobiology, Concordia University, Montréal, QC, Canada; ^4^Département de Génie Informatique et Génie Logiciel, Polytechnique Montréal, Montréal, QC, Canada

**Keywords:** oscillation, phase-locking, cerebellum, rhythmicity, network

## Abstract

Oscillations in the granule cell layer (GCL) of the cerebellar cortex have been related to behavior and could facilitate communication with the cerebral cortex. These local field potential (LFP) oscillations, strong at 4–12 Hz in the rodent cerebellar cortex during awake immobility, should also be an indicator of an underlying influence on the patterns of the cerebellar cortex neuronal firing during rest. To address this hypothesis, cerebellar cortex LFPs and simultaneous single-neuron activity were collected during LFP oscillatory periods in the GCL of awake resting rats. During these oscillatory episodes, different types of units across the GCL and Purkinje cell layers showed variable phase-relation with the oscillatory cycles. Overall, 74% of the Golgi cell firing and 54% of the Purkinje cell simple spike (SS) firing were phase-locked with the oscillations, displaying a clear phase relationship. Despite this tendency, fewer Golgi cells (50%) and Purkinje cell’s SSs (25%) showed an oscillatory firing pattern. Oscillatory phase-locked spikes for the Golgi and Purkinje cells occurred towards the peak of the LFP cycle. GCL LFP oscillations had a strong capacity to predict the timing of Golgi cell spiking activity, indicating a strong influence of this oscillatory phenomenon over the GCL. Phase-locking was not as prominent for the Purkinje cell SS firing, indicating a weaker influence over the Purkinje cell layer, yet a similar phase relation. Overall, synaptic activity underlying GCL LFP oscillations likely exert an influence on neuronal population firing patterns in the cerebellar cortex in the awake resting state and could have a preparatory neural network shaping capacity serving as a neural baseline for upcoming cerebellar operations.

## Introduction

With its systematic structure, the cerebellum possesses inherent modularity supporting the flow of information (Voogd and Glickstein, 1998; Llinás et al., 2004; Ito, 2010), and its coding capacity has been the object of multiple decades of neurophysiological inquiry (Eccles et al., 1967; Ito, 2006; Heck, 2015). Some of the mechanisms uncovered focus on oscillatory activity: one way to control the spatiotemporal flow of information across modules is through interconnected oscillating networks: these can be variably coupled to support information flow according to specific frequencies or multiple modes (Fries, 2015; Maris et al., 2016). In turn, these oscillations can act as a modulator or amplifier of information throughput across and within circuits (Akam and Kullmann, 2010).

Recent reviews highlight the capacity of cerebellar cortex circuits to harbor rhythmic activity, with potential functional roles, including modulating the timing of cerebellar neuronal firing (Isope et al., 2002; D’Angelo et al., 2009; De Zeeuw et al., 2011; Courtemanche et al., 2013). For instance, olivo-cerebellar neurons carry an intrinsic 6–10 Hz intracellular rhythm able to influence the timing of Purkinje cell complex spikes across the cerebellar cortex (Welsh et al., 1995; Lang et al., 1999; Llinás, 2009). The granule cell layer (GCL) also shows local field potential (LFP) rhythmic activity, namely at 10–25 Hz in the monkey (Pellerin and Lamarre, 1997; Courtemanche et al., 2002), and 4–12 Hz in the rodent (Hartmann and Bower, 1998; O’Connor et al., 2002; Dugué et al., 2009). High-frequency oscillations (150–300 Hz) have also been detected in the Purkinje cell and molecular layers, or cerebellar cortex surface (Chéron et al., 2004; Middleton et al., 2008; de Solages et al., 2008; Groth and Sahin, 2015). Finally, slower (<1 Hz) oscillations have been recorded in the cerebellar cortex of rodents (Ros et al., 2009) and tottering mouse (Chen et al., 2009). Overall, these LFP oscillations provide indirect evidence of rhythmic synaptic input that could serve to influence the firing patterns of cerebellar networks, and their temporal coordination, influencing neuronal coding and communication (De Zeeuw et al., 2011; Courtemanche et al., 2013).

Cerebellar cortex GCL oscillations between 4 and 25 Hz are present at rest (Hartmann and Bower, 1998; Dugué et al., 2009; D’Angelo et al., 2009; Courtemanche et al., 2013), and can enhance cerebro-cerebellar synchronization even though these rhythms are in distant structures (O’Connor et al., 2002; Courtemanche and Lamarre, 2005). Rhythms in the 5–30 Hz range have indeed shown capacity to dynamically link distant systems *via* local and long-range neuronal firing and connections (Bullock, 1997; Buzsáki and Draguhn, 2004; Buzsáki, 2006; Senkowski et al., 2008). It is well-established that LFPs are related to the synaptic activity (Buzsáki and Draguhn, 2004): single-unit activity should thus have a role in how GCL LFPs synchronize with cerebral cortex LFPs. However, GCL oscillations do not have a readily defined substrate, though granule and Golgi cells should be implicated, the latter coupled *via* gap junctions (Courtemanche et al., 2002; Maex and De Schutter, 2005; D’Angelo and de Zeeuw, 2009; Simões de Souza and De Schutter, 2011). Indeed, GCL oscillations show a strong relation to granule cell firing (Pellerin and Lamarre, 1997; Hartmann and Bower, 1998; Courtemanche et al., 2002) but the extent of the influence across the layers has not been assessed. Granule cells have rhythm-permissive cellular properties and could be part of a resonant network (D’Angelo et al., 2001, 2009). Intrinsic oscillatory capacities of the GCL local network have been modeled (Maex and De Schutter, 2005; Dugué et al., 2009; Honda et al., 2011; Simões de Souza and De Schutter, 2011; Sudhakar et al., 2017). For instance, Golgi cell-mediated feedforward and feedback loops (Forti et al., 2006; D’Angelo, 2008; Dugué et al., 2009; Galliano et al., 2010), and Golgi-Golgi electrical synapses could be implicated in the rhythm formation (Dugué et al., 2009; Vervaeke et al., 2010; Simões de Souza and De Schutter, 2011; Robinson et al., 2017). Further in the circuit, in a limited dataset, we saw that Purkinje cell simple spikes (SSs) can follow the 10–25 Hz GCL rhythm, contrary to complex spikes (Courtemanche et al., 2002). In contrast, for a slow <1 Hz rhythm, only complex spikes can follow the activity (Ros et al., 2009), and fast Purkinje cell layer oscillations can entrain SSs (Chéron et al., 2004; Middleton et al., 2008; de Solages et al., 2008). It is unclear if this oscillatory activity can influence the cerebellar nuclei, but the synchronization of SSs promotes the downstream activation of cerebellar nuclei (Person and Raman, 2012a,b).

This report focuses on the relationship between cerebellar cortex units recorded using electrodes and tetrodes with simultaneously recorded GCL LFPs in the awake rat, putting a particular focus on unit phase relation and rhythmicity. We recorded Golgi and Purkinje cell SSs and evaluated their firing patterns concerning 4–12 Hz GCL LFP oscillations. We hypothesized that the unit firing would be related to those oscillations and that Golgi firing in the GCL would be more phase-locked to the oscillations than the SSs, principally because of the diverging/converging connections between the GCL and Purkinje cells.

## Materials and Methods

Data for this study were collected at Concordia University (Montréal, QC, Canada), and École Normale Supérieure (Paris, France), using the same rat strain, along with similar recording techniques and analysis parameters.

### Animals and Behavior

Seven (7) male Sprague–Dawley rats (four rats/Charles River, St-Constant, QC; three rats/Institut de Biologie vivarium, ENS, ~400–500 g) were initially handled and habituated to the lab environment. Once implanted with electrodes, they were housed individually on an 8:00 AM to 8:00 PM reversed light/dark schedule. Recording sessions were conducted in a Lafayette Instruments (Lafayette, IN, USA) test chamber or in a custom dark Plexiglas field arena. Rats were kept in the test area for a period of 1–2 h under dim light and quiet conditions. Most rats explored the area for the first few minutes, then calmed down and stayed relatively immobile; they were kept attentive by the experimenter. All animal handling, care, and surgical procedures were following the guidelines of the respective animal national welfare councils and approved by the respective University Animal Research Ethics Committees.

### Surgical Procedures

The surgery and electrophysiological methods were similar to Dugué et al. (2009) and Gao et al. (2011). Briefly, four rats were mounted with a Neuralynx 12-drive electrode holder (Bozeman, MO, USA) for recordings in the posterior cerebellum; three animals were mounted with a custom headstage housing 1–4 quartz tetrodes (Thomas Recording GmbH, Giessen, Germany). Electrode and tetrode shapes and impedances were optimally chosen to capture both LFP and unit signals, and electrodes could be moved longitudinally with precision (described below), permitting to isolate units during the experiment.

Similar procedures were followed for surgery for both sets of animals. Body temperature was continuously monitored with a rectal probe and maintained with a heating pad. General anesthesia was induced either with (1) an i.m. injection of ketamine hydrochloride (Ketaset, 100 mg/kg) and xylazine (AnaSed, 2.2 mg/kg) and maintained by supplemental injections as required; or with (2) a ketamine-xylazine mixture, and maintained with a mixture of isoflurane (0.5–1.5%) and oxygen. All rats were then mounted on a stereotaxic instrument. To reduce bronchial secretions, rats were injected with 0.04 mg/kg s.c. of atropine sulfate before inducing anesthesia. The skull and dura over the posterior cerebellum were removed using a dental drill and precision forceps. The headstage was implanted and fixed in the skull with screws in the frontal and parietal bones above the cerebellar cortex with dental cement. At the end of the surgery, the wound was carefully sutured and covered with antibiotic cream. Animals were allowed to recover several days before recording.

### Electrophysiological Setups and Recordings

Methods for LFP and unit recordings closely followed published procedures (Dugué et al., 2009; Gao et al., 2011). For the multiple single electrode implants, the implantation target was at Bregma −12, lateral 2.5–3, aiming for the crus II/paramedian lobule. One bone screw served as the ground contact and a stainless-steel needle (19 G) placed in brain tissue, providing a large cylindrical contact at the surface of the cerebellum, served as reference. Three to seven tungsten microelectrodes with shank diameters of 75 μM and impedances around 1 MΩ (0.2–1.5 MΩ–FHC Inc., Bowdoin, ME, USA) were inserted into individual drives, mounted onto the headstage. Each microelectrode could be moved independently using a small screwdriver to advance and retract the electrode. LFP data were on-line filtered between 1 and 475 Hz and sampled at 2,003 Hz. For unit activity, the signal was filtered between 600 and 6,000 Hz and sampled at 32 kHz. Spike isolation was achieved by lowering or retracting the individual microdrives (160 μm/turn precision, with usual increments of about $\frac{1}{4}$ or $\frac{1}{2}$ turn, so 40–80 μm, or even less when isolating a unit). Adjustments on spike detection were then performed on the Neuralynx DAS software 32-point digitized thresholded waveform, overlaid to verify reproducibility. Also, the analog unit signal was monitored for waveform stability on an oscilloscope and spike loudspeaker sound output, useful during microelectrode positioning. Mostly one single unit, sometimes two, could be isolated at a site.

For three animals, tetrodes were implanted in a lightweight tetrode headstage holding multiple microdrives, each with a reference and 1–4 quartz tetrodes (constructed as four platinum/tungsten-cores in a quartz rod, sculpted with a sharp tip). The microdrives were moved *via* a cubic screw mounted on a threaded rod. Tetrodes were protected by a stainless steel tube and a 30 G beveled guide tube. Drives were enveloped in a grounded conic piece of cardboard and aluminum foil. The tips of the tetrodes were cleaned and gold-plated to lower their impedance to 0.1–0.3 MΩ. Signals were acquired with a Tucker-Davis Technologies System 3 (TDT, Alachua, FL, USA), filtered at 0.1 Hz to 8 kHz with a Butterworth filter, then differentially amplified, sampled at 25 kHz, and stored to disk for off-line analysis. During tetrode adjustment and recordings, lowered in increments of 10–50 μm, the neuronal activity was continuously monitored through loudspeakers and displayed on a computer screen. To isolate spikes, continuous wide-band extracellular recordings were first filtered off-line with a two-pole Butterworth 500 Hz high-pass filter. Spikes were then discriminated by thresholding the filtered trace and extracting the main parameters of their waveform (width and amplitude on the four channels). LFPs were also extracted from one of the tetrode channels wide-band signal, with the initial signal downsampled at 2,003 Hz and low-pass filtered at 475 Hz.

Recording placement of at least one single microelectrode or tetrode in an animal would be optimized for GCL activity with oscillatory LFPs: this would be monitored on-line, often with phasic multiunit activity serving as a guide. The electrode site would be further adjusted if a putative GCL unit was nearby. The other probes could then be independently moved to seek other units, searching for Golgi unit activity in the GCL (which usually had a moderate firing rate), or sharper cell activity in the neighboring Purkinje cell layer (which usually had a much faster firing rate). The rats were brought to the laboratory for durations of up to 90 min of quiet rest, and the rat would be kept periodically attentive by providing small food pellets and water in the recording chamber. Continuous recording sessions lasted up to 20 min.

### Data Analysis

LFP and unit signal processing and quantitative analyses were performed using NeuroExplorer (Nex Technologies, Littleton, MA, USA) and MATLAB (MathWorks, Natick, MA, USA), the latter with routines based on standardized functions (e.g., signal processing toolbox). LFP periods of strong oscillations, in contrast with periods when oscillations were weaker, were identified using spectrograms, calculated using the discrete short-time Fourier transform to evaluate rhythmicity. A multi-parametric algorithm was used to identify oscillatory periods in the 4–12 Hz band of the spectrogram, corresponding with rodent GCL oscillations from other *in vivo* studies (Hartmann and Bower, 1998; O’Connor et al., 2002; Dugué et al., 2009; Frederick et al., 2014; Robinson et al., 2017). This algorithm has been used previously to detect and process various rhythmic signals [gamma (Lévesque et al., 2009), and theta (Berryer et al., 2016)]. In certain cases, coherence spectrograms were also used to evaluate LFP synchronization. The first step consisted of a spectrogram analysis, where data sampled at 2,003 Hz were decimated by a factor of 15 after being low-pass filtered by a 100th order FIR filter. The spectrogram was then elaborated from the dataset, separated into one-s intervals (134 points) to which a Hamming window was applied, and the windows were overlapped by 50%. The discrete Fourier transforms were evaluated over 256 points with zero paddings. A gamma correction with a factor of 0.2 was applied to the spectrogram to improve contrast with random noise. Following, for each time window, the algorithm identified the peak frequency in the band of interest and calculated the energy within a 1 Hz band centered on this peak. To be considered a valid candidate, a peak must have met time and frequency domain criteria, with parameters adjusted to the analyzed trace. To better describe, here are some example settings for one particular session: the energy within the peak was set at least at 30% of the largest peak within a 60-s window (time-domain criterion), and containing at least 40% of the band energy at that time (frequency domain criterion). This identified an oscillatory period composed of a succession of peaks with a determinate track in time and frequency. In those, the relative peak intensities must not have varied in time by more than 100% per second, successive peak frequencies must not have varied by more than 7.5 Hz per second, and there must have been a continuous track of candidate peaks at least 5 s long, and the oscillatory period had to be longer than or equal to a 3 s duration. These parameters were adjusted for each recording location and session. This would permit detections of periods of oscillation, as shown in the example in Figure 2. To compare the spike-LFP oscillation over several cycles, we opted for a duration threshold that allowed to characterize a strong oscillatory influence, with salient oscillatory periods lasting long enough to potentially indicate a state-like influence on the neurophysiological signal. The parameters were selected through systematic data analysis and had the advantage of standardizing the detection of oscillation events over all data sets, eliminating bias. LFP traces were normalized using a *z-score* transform, and the power spectral density was also normalized to the maximum values in the dataset. The end result was a list of “oscillation periods” throughout the recording file and interspersed in between those, periods of weaker or no oscillation. Figure 2 illustrates detected periods for 50 s of LFPs, delineated with a red box.

**Figure 1:**
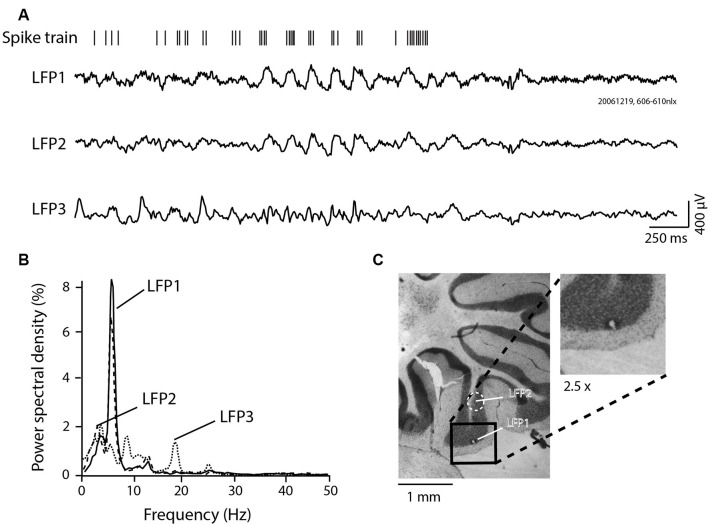
Local field potential (LFP) 4–12 Hz granule cell layer (GCL) oscillations recorded in the posterior lobe of the awake rat. **(A)** GCL activity recorded at three different sites (three LFP traces, LFP1, 2, and 3), with corresponding single unit Golgi spike train recorded at the same site as LFP1. Notice the relative similarity between LFP1 and LFP2, with LFP3 being relatively different. Also, notice the in-phase spiking for the spike trace relative to LFP1. **(B)** Power spectral density results for each LFP shown in panel **(A)**. **(C)** Lesion made in the paramedian lobule GCL, at the site of recording for LFP1, with the relative localization of the LFP2 recording site. Inset: Magnification (2.5×) of the lesion site.

**Figure 2:**
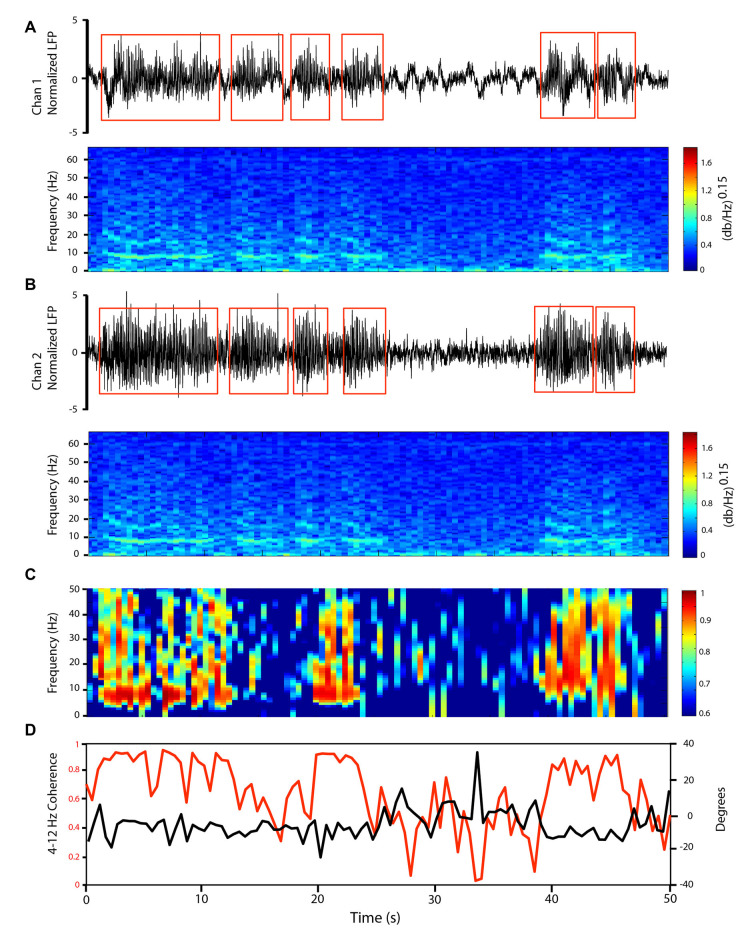
LFP oscillations at 4–12 Hz in the cerebellar cortex GCL show variable coherence patterns across time. Simultaneous GCL LFP recordings from two microelectrodes distanced ~3 mm (Chan 1 in paramedian lobule, Chan 2 in Crus II). **(A,B)** Simultaneously recorded GCL LFPs (top) and corresponding frequency spectrogram (bottom) showing changes in oscillatory activity through time. LFP trace amplitude *z*-score normalized, to perform oscillatory episode detection. Detected episodes of oscillation are represented by the red square box. Frequency spectrogram shown with 1-s windows. **(C)** Coherence spectrogram, 1-s windows, showing 0–50 Hz coherence patterns in time. **(D)** 4–12 Hz Coherence (red line), and corresponding phase lag (black line) between the two LFP traces.

For unit data, the digitized spikes were processed for single-unit identification. For single electrode recordings, this was done in SpikeSort (Neuralynx, Bozeman, MT, USA) in manual mode, focusing on spikes corresponding to an adapted extracellular action potential shape template, estimated from the overlaid spikes (based on >100 detections). Secondary elements were also used such as the 2-D scatterplot distribution of the spike amplitude and duration values (e.g., peak height, valley depth, action potential duration). Interspike intervals (ISIs) of less than 1 ms were removed. Generally, units with fewer than 2% of the ISIs under 3 ms were kept. This methodology is based on parameters used in articles using similar recording techniques (Csicsvari et al., 1998; Stratton et al., 2012; Lévesque et al., 2016, 2018; Chen et al., 2018). For tetrode recordings, a similar process was used; data were hand-clustered by polygon-cutting in two-dimension projections of the parameter space using Xclust [(Davidson et al., 2009) and Matt Wilson, MIT]. Parameter space was centered on spike amplitude and width properties. The quality of clustering was evaluated by inspecting the autocorrelograms of the units. The unit classification was based on electrode tip localization relative to the surface and the GCL (with the characteristic dense background activity), the action potential properties (spike amplitude or presence of a rare complex spike), and the inter-spike interval properties [such as the median ISI vs. median absolute difference (MAD) ISI relation, as presented in Vos et al., 1999]. The indirect nature of this classification makes us qualify our cell types as putative (see “Limitations” section), but similar in properties to previously reported. The database of units is described in “Database of Units” section.

The relation between the timing of single-unit activity and LFPs was established by cross-correlating the spike train with events representing detected LFP peaks (FindPeaks, Tom O’Haver, U. Maryland; which was later included in the MATLAB functions) during oscillatory periods (Courtemanche et al., 2003). The cross-correlation between the LFP peak events and the spike events was calculated, with the LFP peak used as the reference point (Lamarre and Raynauld, 1965; Gerstein, 1999; Courtemanche et al., 2002, 2003). To establish the significance of the spike-LFP relationship, an index was computed based on the cross-correlogram for each unit (Destexhe et al., 1999). We computed artificial controls, using 50 or more artificial spike trains generated with randomized interspike delays (equal number of spikes as the original spike train, so isofrequency). For each artificial spike train, an LFP peak-triggered histogram with mean and SD for each 10 ms bin was processed. Within two cycles on either side (250 ms, corresponding to two cycles at 4 Hz), peaks above or valleys below 2 SD from the mean of the spike-shuffled control histogram were then identified in the LFP-triggered cross-correlogram peaks. LFP rhythmic modulation at this frequency will usually influence multiple consecutive bins; consequently, following this detection, a summation of counts from seven bins, three before the peak, the one on the peak, and three after, gave a “density” count for the detection. Seven 10-ms bins (70 ms) correspond to $\frac{1}{2}$ cycle at 14 Hz, chosen to follow faster-modulated units. This summation was divided by the median count for the overall cross-correlogram to account for the general quantity of collisions, providing values moving about around 1. Finally, a value of 7 was subtracted to resemble a general count per bin, as the sum of 7 bins would have values around 7. We termed this index the phase lock index or PLI. Units with a high PLI would thus show a strong spike-LFP relationship. The spike-LFP phase relationship, for each cell with a significant LFP-triggered histogram peak, was computed using the following equation: phase of peak (rad) = [time of peak (in ms) × 2π] /cycle time (ms; Fisher, 1995; Perez-Orive et al., 2002). This permitted to produce of a polar histogram of the spike–LFP phase relationship that could be generated for the group of units.

In the same way, an algorithm based on the 2 SD shuffling of spikes was also used for determining the significance of the spiking autocorrelation peaks: a rhythmicity index (RI), using the height of the peaks or the depth of the valleys when located outside the 2 SD threshold, adapted from previous methods (Sugihara et al., 1995; Lang et al., 1999). The difference in amplitude between the detected peak/valley and the next valley/peak within half a cycle was calculated (Sugihara et al., 1995); for instance, if a significant peak was detected at 120 ms, we calculated the difference in amplitude between this peak and a valley between 60 and 180 ms. The summation of peak/valley distances (that were different from the shuffled values) permitted to calculate the RI; this value is above zero then permitted to define if the cell was oscillatory.

### Histology

After the last recording session, electrolytic lesions (200 μA, 45 s, anodal) were made in the cerebellar cortex at selected sites where oscillations of single units were found, while rats were under ketamine-xylazine anesthesia. Two days later, rats were deeply anesthetized with ketamine-xylazine and perfused through the heart using a buffered 10% formalin–0.9% saline solution. The brains were removed and kept in 10% formalin for at least 48 h. They were then put in a 20% sucrose-formalin solution for another 48 h. The brains were frozen in pulverized dry ice and then sliced in a cryostat. The 40-μm thick sections were mounted on glass slides coated with gelatin. The slides were stained using a Cresyl Violet solution. The location of the lesions was evaluated following the denomination in the Paxinos and Watson (1998) atlas and electrode tracks were reconstructed for localization of recording sites.

## Results

### Cerebellar Cortex GCL Oscillations in the Awake Rodent

Figure 1 presents Golgi cell spike activity and simultaneously recorded LFPs at three different sites in the posterior lobe cerebellum (three LFP traces, LFP1–3, Figure 1A). For each trace, the corresponding power spectral density signal was computed and is shown in Figure 1B. For LFP1, single-unit activity simultaneously recorded with the LFP is shown. For this experiment, the location of electrode #1, corresponding to LFP1, was marked by an electrolytic lesion (Figure 1C, and inset). The other recording site from a nearby electrode sharing a similar track, LFP2, is also indicated on the histological section, located 0.96 mm above the lesion, in the GCL or the GCL-white matter border. Both LFP1 and LFP2 were thus recorded in the paramedian lobule. LFP3 was recorded in a different plane, at a similar depth as the lesion, yet also in the cerebellar cortex from the neighboring multiunit activity, likely in the anterior copula of the pyramis region (not shown). As can be seen from the figure, the simultaneously recorded LFP activity differed at the three individual recording sites. LFP1 and LFP2 appear more similar: these were closer and presumably both in the GCL. A period of oscillatory activity is evident around the midpoint of the recording trace, for LFP1 and LFP2. LFP3 was not oscillatory in the 4–12 Hz range. The power spectral density analysis in Figure 1B confirms the similar oscillations on LFP1 and LFP2. Golgi unit activity simultaneously recorded with the LFP1 oscillations showed bursts occurring in-phase with the oscillation cycles.

As another way to approach the local nature of the LFP, we provide an example of the effect of the presence of oscillations on cerebellar inter-electrode synchrony. The GCL oscillations could synchronously affect neighboring electrodes: Figure 2 shows recordings from two electrodes within the posterior lobe GCL, one (Chan l) in the paramedian lobule, and the second (Chan 2) in Crus II. The 4–12 Hz LFP oscillations showed waxing and waning qualities at ~8 Hz on both channels, as seen on the power spectral density spectrogram (Figures 2A,B). Our detected periods (see “Materials and Methods” section) with strong 4–12 Hz oscillations are shown on the two LFP channels. In many instances, multiple channels could show simultaneous oscillations. In optimal conditions, these oscillation periods could last several seconds (Figure 2). Detected oscillation periods (which had to last longer than 3 s) would last on average 4.5 s, and on average would be present 20.9 ± 13.0% of the total recording time. During oscillations periods, the 4–12 Hz oscillations could be synchronized between the two traces, as can be seen on the coherence spectrogram (Figures 2C,D). To illustrate, we show a 50-s recording example with two electrodes in the rodent awake resting cerebellum. From the 4–12 Hz coherence spectrogram (Figures 2C,D), in the presence of oscillations, coherence values would reach 0.9 for long periods (e.g., 40–48 s). During these periods of stronger coherence, the phase relationship between the two traces was around −10 degrees, so close to in-phase. In out-of-oscillation periods (e.g., 25–40 s), the coherence would drop markedly, with accompanying phase variations. To further document the effect of oscillations on coherence, we analyzed the alpha/theta coherence between electrodes in three of our rats, with three sessions each, in a dataset of over 5,000 detected periods with at least one electrode with detected oscillations, and an overall pool of over 28,000 time periods (see Supplementary Data). We saw that when oscillations are present on at least one electrode, there would always be an increase in coherence. As this oscillatory phenomenon can serve to describe network coherence in the cerebellar cortex, we next investigated how LFPs were related to unit firing, providing an indirect but useful view of the effects of rhythmic synaptic inputs on specific neuronal groups.

### Database of Units

A total of 207 cells were isolated with the microelectrodes and tetrodes, and recorded simultaneously with the LFPs, and were classified by neuronal type. Of these, we managed to capture 115 stable cells with the simultaneous presence of 4–12 Hz LFP oscillations. Descriptive data on our sample is given in Table 1, and the classification is further detailed below.

**Table 1:** The number of single units recorded and classified in the Golgi and Purkinje cell simple spike (SS) groups.

Group	*n*	Firing rate (spikes/s)	Median ISI (ms)	Units with simultaneous 4–12 Hz
				LFP oscillations
Golgi	46	7.1 (± 5.2)	72.7 (± 36.8)	74% (34/46)
Purkinje cell SS	126	40.6 (± 24.1)*	24.6 (± 12.7)	64.3% (81/126)

We show the average firing rate and the average median ISI (±standard deviation). Approximately two-thirds of the unit sample recorded in each group had simultaneous 4–12 Hz LFP oscillations (*t-test, *p* < 0.05, Golgi vs. SS).

**Figure 3:**
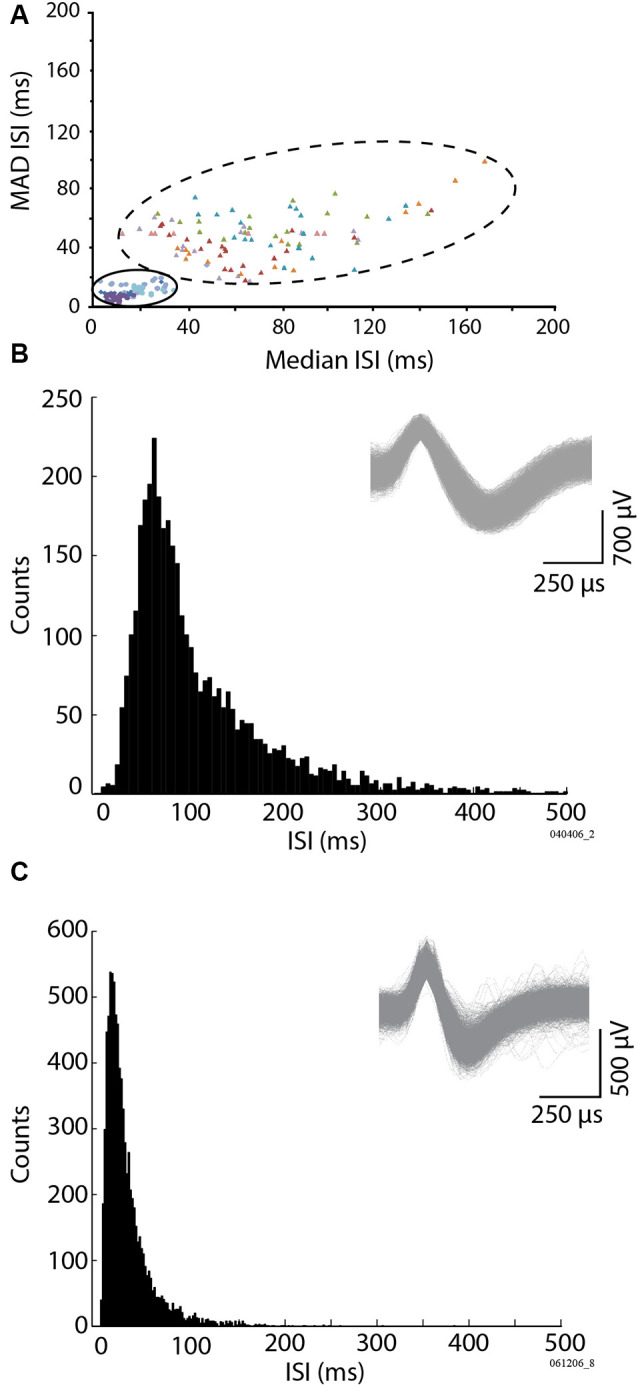
Identification of the Golgi cells vs. Purkinje cell simple spikes (SSs), based on the firing properties. The method follows the one used by Vos et al. (1999), based on the variability of the cell firing (MAD ISI) vs. its median firing values (Median ISI). **(A)** Representation of two subpopulations of units, Golgi cell units (large ensemble, filled triangles, six cells, colors identify different units), and Purkinje cell SSs (small ensemble, filled circles, and diamonds, four cells), by the relationship between their median inter-spike interval (Median ISI), and the absolute deviation of their median firing inter-spike interval (MAD ISI). **(B)** Inter-spike interval of a representative identified Golgi cell. **(C)** Inter-spike interval of a representative Purkinje cell SS. **(B,C)** Insets: example spike waveforms for a Golgi cell **(B)** and a Purkinje cell SS **(C)**.

The identified single units had variable extracellular firing properties. During our recordings, these were initially classified based on: (1) the location of the isolated cell concerning the track and the background activity (i.e., the cerebellar cortex layer), with the typical GCL dense multiunit activity, or the sparser sharp fast-spiking Purkinje cell SSs; and (2) the action potential shape and duration, along with the rare co-occurrence of the occasional complex spike for Purkinje cells. Because our approach focused on GCL oscillations, it should be noted that our search for units was GCL-centric, and we did not seek out complex spike recordings. Figure 3 displays certain typical spike firing characteristics of the units. To further refine our classification, we also used the offline identification method of Vos et al. (1999), which graphically compares the median of the inter-spike interval and the median absolute difference of the ISI (MAD ISI) to identify the groups of spikes corresponding to a given cell type. The median ISI was calculated for 20 consecutive bins of 10 spikes. For the same 20 consecutive bins, the MAD ISI representing the median difference between each ISI and the median ISI was obtained. Figure 3A provides the clustered distribution of a representative sample of the Golgi and Purkinje cell types. Evident are the differences between our two identified subpopulations of spikes: the two-dimensional spread of the Golgi spike values is more spread out, while the Purkinje cell SS values were all aggregated towards the graph origin (Figure 3A). A representative ISI histogram for a Golgi spike is shown in Figure 3B, while the equivalent for a Purkinje cell SS is shown in Figure 3C. Using those methods, out of 207 recorded isolated units, spikes were classified as coming from either putative Golgi cells (*n* = 46) or Purkinje cells SSs (*n* = 126). The remaining 35 isolated cells were classified as coming from either Purkinje cell complex spikes (*n* = 3, easily identified larger spikes with after-ripples coming from Purkinje cell layer or molecular layer), mossy fibers (*n* = 8) or were classified as isolated spikes from unidentified cells (*n* = 24), as their respective 2-D median ISI vs. MAD ISI distribution was different from typical Purkinje cell SSs or Golgi cell spikes. Overall, our Golgi cells had a mean firing rate around 7 Hz (see Table 1) and a median ISI in the range of the samples described in Vos et al. (1999) and Holtzman et al. (2006). Purkinje cell SSs we recorded had a mean firing rate of around 41 Hz. As our sample was better defined for the Golgi cells and Purkinje cell SSs, we decided to focus on these two groups for the rest of the analysis. The firing rate between the Golgi cells and Purkinje cell SSs was significantly different (*t*-test, *p* < 0.05).

### Spike-LFP Relationship

**Figure 4:**
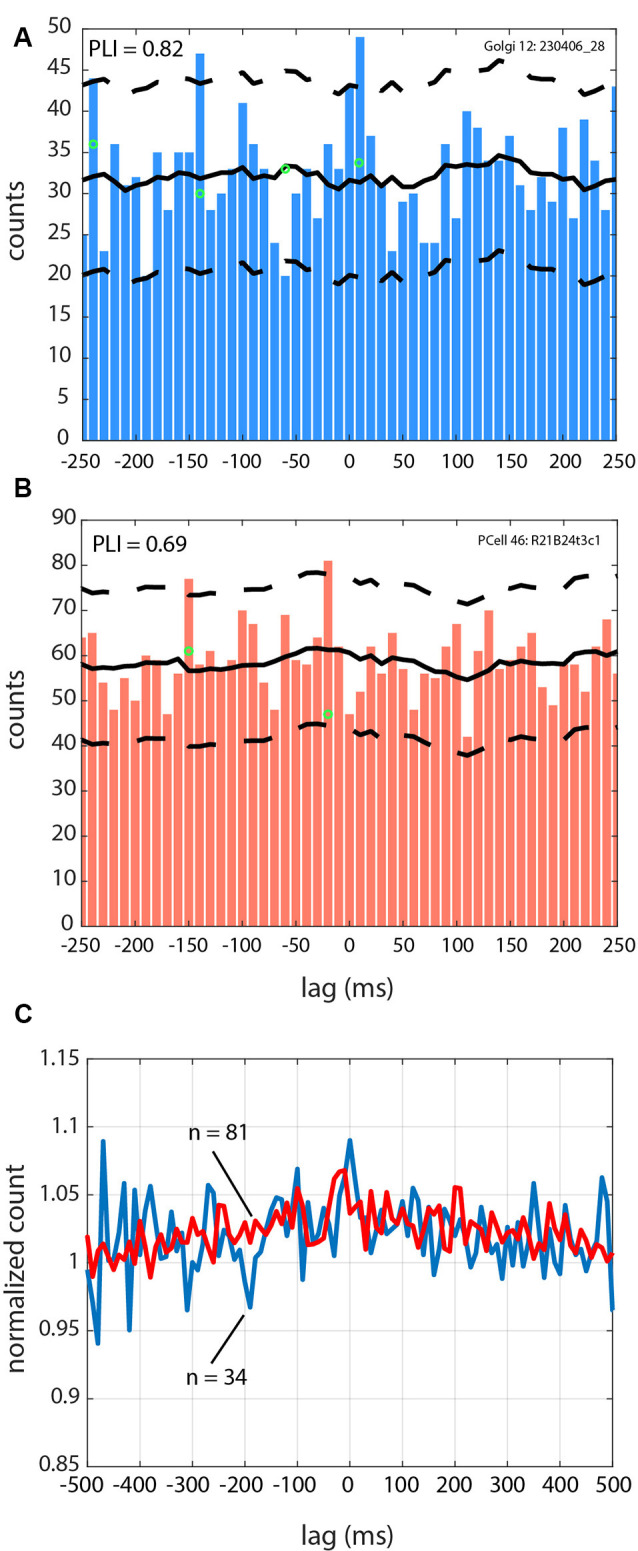
Spike-LFP relationships for Golgi and a Purkinje cell during 4–12 Hz oscillatory LFPs. **(A)** LFP-triggered spike histogram for a Golgi cell, with a peak at +10 ms. **(B)** LFP-triggered spike histogram for a Purkinje cell SS, with a peak at −20 ms. **(C)** Averaged trace for the cells in the Golgi cell group (blue line), and the Purkinje cell SS group (red line). The averaged trace has been normalized across all bins to a value of 1 so they can be superimposed. For **(A–C)**, zero is the time of the LFP peak. For **(A,B)**, the average of the spike-shuffled control is shown with the full black line, and the ±2 SD is indicated with the dashed black line.

We analyzed if the spikes coming from the different cells followed a specific firing pattern relative to the simultaneously recorded LFP oscillations. Of the overall sample, 34/46 Golgi cells were recorded with simultaneous LFP oscillations, and the same for 81/126 Purkinje cell SSs (see Table 1). Overall, using the spike-shuffled analysis to determine a phase-locking index or PLI, we determined that 74% (25/34) of Golgi cells and 54% (44/81) of Purkinje cell SSs had some degree of phase-locking with the simultaneous 4–12 Hz LFP oscillations (see Table 2). The average value of the PLI for Golgi cells was 0.65 (± 1.1; median of 0.36), while for the Purkinje cell SSs, the PLI had values of 0.32 (± 0.6; median of 0.10). Examples of LFP-triggered histograms (spike-LFP cross-correlograms) for a Golgi cell and Purkinje cell SS are given in Figure 4, showing a strong relationship for both. The Golgi cell would fire preferentially in-phase with the peak of the LFP (lag of +10 ms for this cell, PLI = 0.82, Figure 4A), while in another recording, the Purkinje cell SS would also fire close to in-phase (lag of −20 ms, PLI = 0.69, Figure 4B). For both LFP-triggered histograms, the modulation around the peak can be seen relative to the shuffled 2 SD thresholds. As is done customarily, the identified peak (or valley) was the one closest to zero lag, where the temporal relationship of the cross-correlation is the clearest, working on short timescales (Lamarre and Raynauld, 1965; Perkel et al., 1967; Frölich, 2016). When attempting to see if the phase would be matched at a group level, the averaged LFP-triggered counts for Golgi cells and Purkinje cell SSs (normalized to their average value across all bins) show only a weak modulation around the 0-time lag (Figure 4C). An implication is that these cell-LFP relationships could be different across cells, requiring a better-adapted method to capture the group response.

**Table 2:** Properties of the two groups of units recorded, in phase-locking and in rate.

Group	Units with simultaneous	Units with phase	Firing rate (spikes/s) for	Firing rate (spikes/s) for
	LFP oscillations	relation (PLI > 0)	units with PLI > 0	units with PLI = 0
Golgi	34	73.5% (25/34)	6.7 (± 4.7) median: 5.8 (*n* = 25)	8.5 (± 6.5)^ns^ median: 5.0 (*n* = 9)
Purkinje cell SS	81	54.3% (44/81)	30.5 (± 18.0) median: 23.8 (*n* = 44)	53.5 (± 24.4)^∧^ median: 44.2 (*n* = 37)

*We show the number of isolated units with a significant relationship with oscillatory LFPs, as defined by the phase-locking index (PLI) for the LFP-triggered cross-correlation. The average firing rate (spikes/s) was different between the Golgi cell and the Purkinje cell simple spike samples (Pcell SS, *t*-test *p* < 0.05). The firing rate for the Golgi cells between phase-locked and non-phase locked units was not different (K–W test, ns), Purkinje cells simple spikes firing rates did, however, show a difference concerning the phase-locking (^∧^K–W test, *p* < 0.05)*.

**Figure 5:**
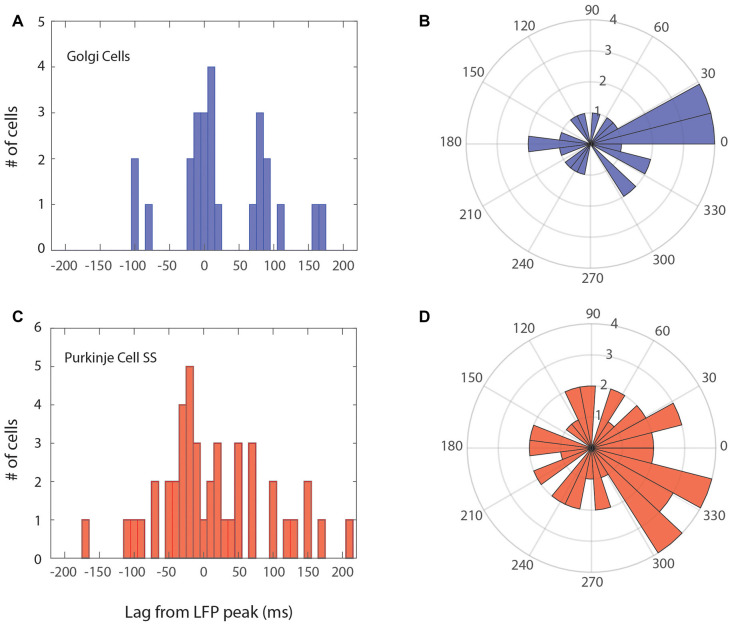
Phase relationship for Golgi cells and Purkinje cell SSs within the LFP oscillation cycle. The main peak of the LFP-triggered histogram is taken to represent each cell. Left **(A,C)** peak alignment in the time domain; Right **(B,D)** angular (phase) relation, centered on the peak of the LFP (zero). **(A,B)** Relation of Golgi cells firing vs. the LFP cycle. Overall, the Golgi cells were more phase-locked with the peak of the LFP cycle or previous/subsequent cycles. **(C,D)** Relation of Purkinje cell SSs vs. the LFP cycle, who had more phase-locked cells with the ascending phase towards the peak, however, in a more variable manner.

As a more precise way to assess the phase relation for the population, cells that were phase-locked were represented according to their peak in the LFP-triggered histogram. This can be seen in Figure 5, with the phase-locking peak in the time domain relative to the peak of the LFP, which was also converted to an angular distribution. The temporal distribution for the 25 Golgi cells is shown in Figures 5A,B, while the one for the 44 Purkinje cell SSs is given in Figures 5C,D. These graphs show a modulation of the spiking activity throughout the cycle, and the preferred phase for the units. For the Golgi cells, the units tended to discharge mostly in phase with the peak of the LFP (around the 10° angle for phase), which can also be seen in the time domain. As for the Purkinje cell SSs, the distribution is slightly more spread around, but it shows an overall tendency to fire during the up-phase of the cycle, close to the peak (circa 315°). The time-domain histogram shows a greater spread around the LFP peak than Golgi cells. These results imply that the Golgi cells and Purkinje cell SSs that are phase-locked with the 4–12 Hz LFP oscillations show a general tendency to fire around the peak of the LFP.

**Figure 6:**
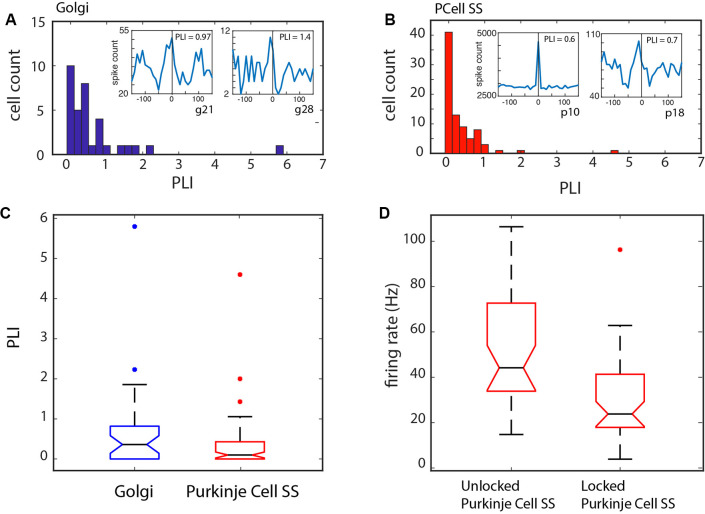
Phase-locking over the samples of Golgi and Purkinje cell SSs. **(A)** Phase-locking index (PLI) distribution for the Golgi cells. Inset: two examples of Golgi cell LFP-triggered histograms. **(B)** PLI distribution for the Purkinje cell SSs. Inset: two examples of Purkinje cell SS LFP-triggered histograms. For both groups, the distribution is skewed towards lower values. A PLI > 0 was our criterion for phase locking. **(C)** Statistical difference between the two groups showing a higher PLI for the Golgi cells. **(D)** Relationship of phase-locking with Purkinje cell SS firing rate. Cells that were phase-locked with the LFP showed a slower firing rate than those that were not.

We also looked at the distribution of the PLI values; for both types of units, it was clear they were not normally distributed (see Figures 6A–C), with the PLI skewed towards lower values. Certain units showed phase-locking (PLI > 0) for values less than 1, and as evidenced by the examples in Figure 4, and the insets in Figure 6, the modulation was appreciable. A comparison between the Golgi PLI and the Purkinje cell SS PLI revealed that the Golgi PLI was significantly higher (Kruskal–Wallis test: *χ*^2^ = 5.54, *p* = 0.0186, df = 1, see Figure 6C).

When comparing the firing rate properties for the units that were phase-locked vs. those that were not, a few differences can be noted. There was again a difference in firing rate between the Golgi cells and the Purkinje cells SSs (Kruskal–Wallis: *χ* = 62.6, *p* < 0.0001, df = 1). Also, the firing rate for the phase-locked and non-phase-locked units was compared: for the Golgi cells, no firing rate difference could be noted (Kruskal–Wallis: *χ*^2^ = 0.32, *p* = 0.57, df = 1), while for the Purkinje cell SSs, the cells that were phase-locked showed a significantly slower firing rate than those that were not (Kruskal–Wallis: *χ*^2^ = 21.32, *p* < 0.0001, df = 1; see Figure 6D). This shows that the slower Purkinje cell SS firing could be better synchronized with the LFP rhythm while the firing rate did not limit phase-locking for Golgi cells. This frequency-specific capacity could be related to the cell’s properties in following a local network resonance mechanism. The specific firing rates are given in Table 2.

### Spiking Rhythmicity

**Figure 7:**
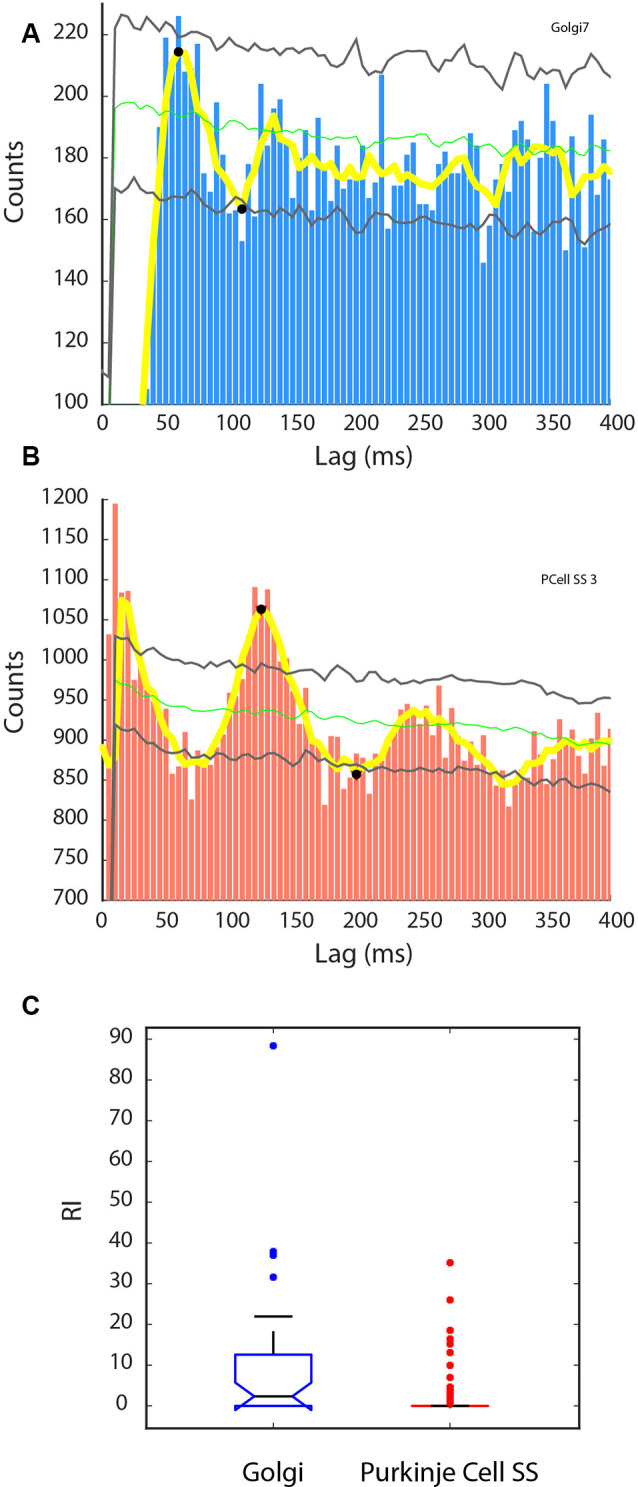
Rhythmic activity in Golgi cells and Purkinje cell SSs. A rhythm index (RI) was calculated based on the unit’s autocorrelogram, from the significant peaks and valleys exceeding the shuffled control for each cell. **(A,B)** Autocorrelograms for a sample Golgi cell (**A**, blue), and Purkinje cell SS (**B**, red). Also indicated are the mean of the shuffled control (green line) ±2 SD (gray lines). Yellow line: running average of the histogram. Black dots signify peaks higher and valleys lower than the shuffled control variability. **(A)** Golgi cell showing a 16.7 Hz rhythm. **(B)** Purkinje cell SS showing an 8 Hz rhythm. **(C)** Statistical difference between the two groups in RI, with the Golgi cell group showing larger RIs than the Purkinje cell SS group.

As a significant proportion of units were found to be phase-locked with the LFP oscillations, it was also interesting to evaluate if the units had a rhythmic discharge. We did so by calculating the rhythm index (RI), based on the unit’s autocorrelogram. Many units showed a rhythmic autocorrelogram, and examples are given in Figure 7. Some Golgi cells showed a rhythm in the 20 Hz range as the example in Figure 7A illustrates (period = 60 ms, for a rhythm of 16.7 Hz). Overall, 17/34 (50%) of our Golgi cells showed a RI > 0, and the RI overall for the Golgi cells was not normally distributed and had a median of 2.35. For the Purkinje cell SSs, 20/81 (24.7%) had a RI > 0, and the example is shown in Figure 7B shows a rhythmic cell (period = 125 ms, 8 Hz); their distribution was also strongly skewed to lower values, with a median set at 0. Comparing the distributions, it is clear that the RI was higher for the Golgi cells than for the Purkinje cell SSs (Kruskal–Wallis: *χ*^2^ = 11.83, *p* = 0.0006, df = 1). Figure 7C shows this disparity. Overall, this analysis shows that the Golgi cells had a greater tendency to show rhythmic properties.

**Figure 8:**
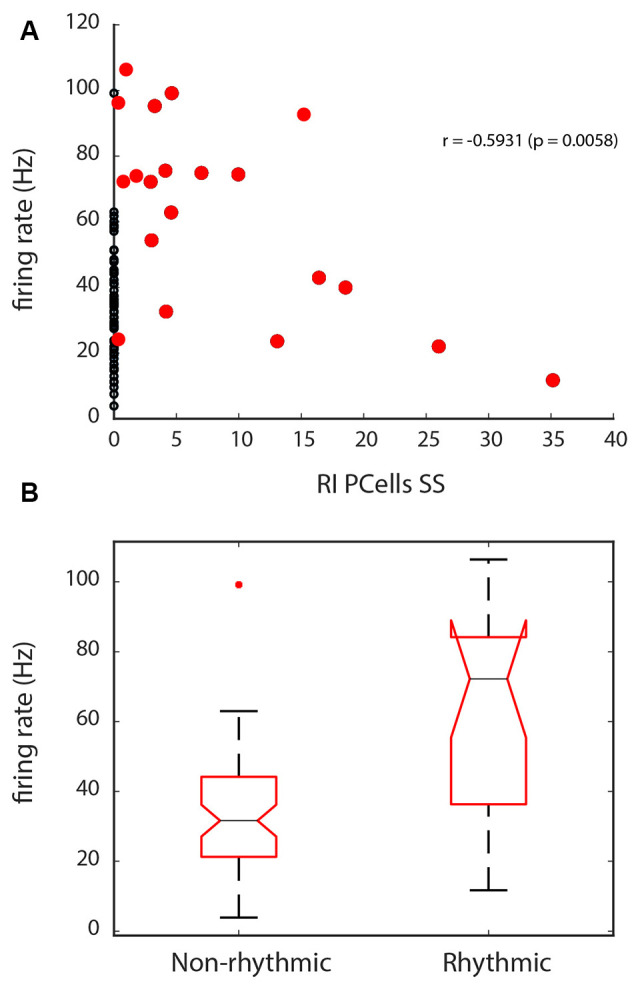
Firing rate properties for Purkinje cell SSs in relation with the Rhythm Index (RI). **(A)** Correlation between the firing rate and the RI, for the units with a RI > 0 (red dots). Units with a RI = 0 are indicated with black dots. Note the inverse correlation with the RI, with lower rate spiking being related to higher RIs. **(B)** Statistical difference in firing rate seen between units with a RI = 0, and those that have a RI > 0. Overall, because of their lower range of firing rate, Purkinje cell’s SSs with no rhythmicity show slower firing.

Finally, we also explored if different factors would better predict which cells would have a greater rhythm index. For Golgi cells, there did not seem to be specific predictive properties, as firing rate or phase-locking did not seem to predict which cells were rhythmic. However, for Purkinje cell SSs, the firing rate was inversely related to the rhythm index, for cells that had a RI > 0 (see the correlation in Figure 8A, with the correlation values for the red dots, *r* = −0.59, *p* = 0.006). This means that cells with a higher firing rate would show a lower RI. Looking at the comparison from the opposite perspective, when we compare the firing rate for Purkinje cell SSs with no rhythmicity (RI = 0), from those with some rhythmicity, the firing rate shows lower values for the non-rhythmic units than for the rhythmic units (Kruskal–Wallis: *χ*^2^ = 14.45, *p* = 0.0001, df = 1, see Figure 8B), which can mostly be attributed to the larger range of firing rates shown across the group of rhythmic units. This can be interpreted as a potential rhythmic influence on the units to increase the firing rate variability for Purkinje cell SSs.

## Discussion

We show here that cerebellar Golgi cells and Purkinje cell SSs can show phase-locking activity with GCL LFP oscillations in the 4–12 Hz frequency range. The phase relationship for both the Golgi and Purkinje cell SSs was mostly around the peak of the LFP. The proportion of those cells that were phase-locked was greater for the Golgi cells than for the Purkinje cell SSs; this can be related to the optimal LFP oscillatory recordings being closer to the Golgi cells—within the GCL. However, the capacity to affect Purkinje cell SSs provide evidence of the capacity of GCL LFP oscillations to influence further elements of the cerebellar cortex networks. It seems fairly clear that the capacity of synaptic input that would stem from the 4–12 Hz rhythmic influence, as seen through the LFP oscillations, does not forcefully drive the Golgi cells or Purkinje cells to fire on each beat. The rhythmic synaptic input likely has a modulatory influence, influencing Golgi and Purkinje cell firing, even if not on every beat. LFP 4–12 Hz oscillations also show potential penetrability across the layers of the cerebellar cortex and have some predictive capacity in determining the timing of the firing of multiple units across the cerebellar cortex of the awake rodent.

### GCL and Golgi Firing Under a 4–12 Hz Oscillatory Influence

Our results show that many Golgi cells tended to follow the 4–12 Hz LFP oscillations in a phase-specific way and that for the overall population, the tendency was to be aligned with the peak of the LFP. These 4–12 Hz oscillations in the rodent are best recorded in the GCL (Hartmann and Bower, 1998; O’Connor et al., 2002), as are the 10–25 Hz cerebellar oscillations in the primate (Pellerin and Lamarre, 1997; Courtemanche et al., 2002). This layer specificity is such that during exploration and positioning of the microelectrodes, the oscillatory LFP signal corresponds well with multiunit firing in the GCL, as can be heard through the audio monitor when playing unit activity. This GCL multiunit activity, presumably coming from a combination of mossy fiber activity and granule cell firing is well correlated with the oscillatory epochs (Hartmann and Bower, 1998; Courtemanche et al., 2002). Our results show that Golgi cell firing is also related to these oscillations, potentially being triggered by an oscillatory afferent drive, and/or contributing to granule-Golgi resonance (Dugué et al., 2009; Robinson et al., 2017).

Golgi cells receive excitatory afferent input from mossy and parallel fibers (Llinás et al., 2004). This excitation gets to the Golgi cell through a feedforward inhibitory circuit (mossy fiber—Golgi cell) and a feedback inhibitory loop (mossy fiber—granule cell/parallel fiber—Golgi cell; Bell and Dow, 1967; Llinás et al., 2004). Both these circuits have potential resonance properties. Modeling has shown that the GCL does have 5–30 Hz resonance capacities (Maex and De Schutter, 2005; Dugué et al., 2009). These circuits constitute a potential mechanism for the Golgi cell phase locking to the LFP oscillations. Overall, this oscillatory pattern could correspond to a pattern of organization of the granule cells-Golgi cells network (Maex and De Schutter, 1998; D’Angelo et al., 2009, 2016), where GCL activity could gate oscillations, and perform group selection for resonance in the layer (Sudhakar et al., 2017). As such, GCL LFP oscillations in the 4–12 Hz range provide evidence of temporal windows of synaptic afferent input during which Golgi cell excitability could be enhanced, in agreement with Dugué et al. (2009).

Also, this pattern of activity could be enhanced by intrinsic properties of elements in the GCL, as both Golgi cell and granule cell-intrinsic properties could support these oscillations. Golgi cells provide rhythmic inhibition on granule cells and have pacemaking activity in the theta frequency range, with resonance for input frequencies of 4 Hz (Dieudonné, 1998; Forti et al., 2006; Solinas et al., 2007). The amount of synaptic noise *in vivo* might obscure the rhythm-generating capacity of Golgi cells; however, they are capable of responding to rhythmic input particularly well (Solinas et al., 2007). In our case, we also found some evidence of rhythmic firing in Golgi cells, as has been shown previously *in vivo*: in the awake animal (Edgley and Lidierth, 1987), in the anesthetized animal (Maex et al., 2000; Volny-Luraghi et al., 2002), with some studies showing strong rhythmicity (Vos et al., 1999; Huang et al., 2014). Golgi cell firing might thus follow network rhythmicity, even if skipping a few cycles; this skipping might be explained by the synaptic noise that prevents reaching the firing threshold in a synchronized way, while the membrane potential can follow baseline rhythmicity (Dugué et al., 2009). Golgi cell firing rhythmicity might require specific network conditions, such as synchronized afferent parallel fiber input (Maex et al., 2000). Also, their capacity to be electrically coupled would greatly influence the formation of Golgi populations following the rhythm (Dugué et al., 2009; Robinson et al., 2017). Granule cells also show specific properties of resonance at slow (best: ~9 Hz) frequencies (D’Angelo et al., 2001), and their responsiveness to input is partially controlled by calcium conductances, modulating their firing rate (Gall et al., 2005). By controlling granular oscillations, Golgi cells could influence the spatio-temporal organization of information processing and storage in the GCL (D’Angelo, 2008; D’Angelo et al., 2009; Sudhakar et al., 2017) as the overall issue of the timing of population activity in the cerebellum gains increased interest (Bareš et al., 2019).

### Extending Further Into the Cerebellar Cortex: Purkinje Cell Simple Spike Firing Under a 4–12 Hz Oscillatory Influence

As many Purkinje cell SSs were also phase-related to the oscillations, the 4–12 Hz oscillatory phenomenon could also influence neurons outside of the GCL. The proportion of Purkinje cells showing this influence is smaller than Golgi cells, but they do show potential “penetrability” of the 4–12 Hz oscillations up to the Purkinje cell layer. Under the strong oscillatory influence, units in the GCL and the Purkinje cell layer activity could fire in relation to the oscillation. In contrast with a network serving basic attentive immobility behavior, during movement, Purkinje cell SSs are related to sensorimotor parameters (Lamarre and Chapman, 1986; Thach et al., 1992; Heck et al., 2007); perhaps the oscillatory synaptic influence we witness through LFPs could provide baseline conditions for forming action-related networks. Information flow between the GCL and Purkinje cell layers has been established (Santamaria et al., 2007): while interneurons like Golgi, unipolar brush, and Lugaro cells influence GCL output to Purkinje cells (Barmack and Yakhnitsa, 2008), a spatio-temporal process must operate to ensure a coordinated activation of SSs. The GCL capacity to excite the Purkinje layer in such a coherent fashion could be due to the mossy fiber arrangement going to Purkinje cells, namely, those coming from the ascending portion of the granule cell axon (Llinás et al., 1981; Gundappa-Sulur et al., 1999; Isope and Barbour, 2002; Lu et al., 2005), or *via* the spatial arrangement of the modulatory connections from the Golgi and Lugaro cells (Barmack and Yakhnitsa, 2008; Sillitoe et al., 2008). These connections could support a coherent temporal representation between the GCL and the Purkinje cell layer within circumscribed cerebellar zones. During GCL oscillations at rest, a coherent sagittal pattern of organization emerges (Courtemanche et al., 2009), which could potentially influence zonal organization at the level of the Purkinje cell layer: through strong anatomical and physiological evidence, the latter has shown heavy parasagittal modularity (Herrup and Kuemerle, 1997; Lang et al., 1999). In the case of our own Purkinje cell SS recordings, we compared GCL oscillations with Purkinje cell firing from a nearby electrode (e.g., from the same guide cannula, thus corresponding to the same sagittal and coronal location). This certainly would favor the phase-locking of Purkinje SS to GCL oscillations.

### Extending Further In and Out of the Cerebellum

The oscillatory entrainment of the cerebellar cortex output cells also opens up the search for the influence outside of the cerebellar cortex and cerebellum, and we speculate on a few mechanisms here. Cerebellar inactivation influences patterns of rhythmic activity in the cerebral cortex (Popa et al., 2013), and Purkinje cell SS can be timed with cortical rhythms (McAfee et al., 2019). More specific to the cerebellar circuits, there are also examples of activity of rhythmic SS firing (Huang et al., 2014). It would be interesting to see how this relates to cerebellar nuclei activity. Indeed, synchronized Purkinje cell activity promotes the downstream activation in cerebellar nuclei (Person and Raman, 2012a,b). The particular “pauses” in the Purkinje cell firing to the deep cerebellar nuclei could be facilitated by the 4–12 Hz rhythm across the cerebellar cortex (De Schutter and Steuber, 2009), favoring synchronicity of firing towards the nuclei (Jaeger, 2011). Varying between 100 and 200 ms long, these pauses relate well with an underlying 5–10 Hz cerebellar cortex rhythmicity (Alviña et al., 2008), and they complement the pacemaker regularity of Purkinje cell firing, which have an important role in the coordinated circuitry (Walter et al., 2006). Besides, the 4–12 Hz rhythmicity also fits well with a recovery time constant of the channels CaV3.1 in those same neurons around 100 ms (Iftinca et al., 2006; De Schutter and Steuber, 2009; Tadayonnejad et al., 2010). Importantly as well, mutant mice that are without the calcium-sensitive BK channels in their Purkinje cells show strong firing rhythmicity in SSs (Chéron et al., 2009) but also in the deep cerebellar nuclei, in the beta range, showing a transmittable rhythmic influence (Chéron et al., 2018). Together, these elements paint a picture that a rhythmic influence could coordinate the activity in the overall cerebellar circuitry under certain conditions, such as in movement preparation (Courtemanche et al., 2013).

### Comparison With Other Cerebellar Cortex Slow Oscillatory Phenomena

Purkinje cell SS firing has been found to adapt to excitability state modulation and slow oscillations, including in a bistable manner (Loewenstein et al., 2005; Chen et al., 2009; Ros et al., 2009). This bistability in the awake animal has been questioned (Schonewille et al., 2006), but could represent a mechanism influencing the firing patterns of Purkinje cell SSs. A depolarized state would favor the firing of the Purkinje cell SSs, and the state-switch from a hyperpolarized state could stem from afferent input/climbing fiber firing. Slow cerebellar oscillations which are around or less than one Hz could also affect cerebellar cortex firing (Chen et al., 2009; Ros et al., 2009). Slow oscillations in the cerebellar cortex of the anesthetized rat (~1 Hz) and awake mouse (~2–5 Hz) recorded by Ros et al. (2009) are tightly synchronized with the cerebral neocortical up-states and promote phase-related firing of Golgi cells, granule cells, and Purkinje cell complex spikes, but not for Purkinje cell SSs. It is unclear if slow and the 4–12 Hz cerebellar oscillations are related. Even slower oscillations (<0.1 Hz) have been recorded in the paramedian and Crus II lobules of the tottering mouse using optical imaging, which was related to Purkinje cell firing (Chen et al., 2009). The co-occurrence of these oscillatory processes, in various anesthetized and awake states and across species, has not been established. Also, as we find here that Purkinje cells SSs can show phasic relations with the GCL 4–12 Hz LFP oscillations, a comparison with the well-established olivocerebellar rhythms at similar frequencies (Lang et al., 1999; Llinás, 2009) would indeed be interesting (Courtemanche et al., 2013). An adapted methodology would have to be crafted to make a direct comparison; our small sample of Purkinje cell complex spikes, as well as our methods, could not allow for population-level analysis for olivocerebellar activity concerning GCL LFPs. Similarly, a comparison with faster oscillatory phenomena in the Purkinje cell layer would also warrant a specific methodology (Servais and Chéron, 2005; de Solages et al., 2008; Middleton et al., 2008).

### Limitations

This study of course has certain limitations. In this study, we did not micro-map the local circuits, which would have required a denser arrangement of electrodes or recording channels (Buzsáki et al., 2012). This would have informed on the more exact span of coherence of our recorded GCL LFP oscillation and potential effects on units. Also, our methodology for determining the classification of units was based on indirect evidence, making our classified units putative Golgi cells and putative Purkinje cells. For Purkinje cells, it is customary to confirm SS identity with the co-recording of complex spikes (Welsh et al., 1999; Gao et al., 2011), which we did not systematically do here, focusing on obtaining both strong GCL LFP oscillations and well-isolated units. For any type of cell, the method of juxtacellular labeling using micropipettes is also quite advantageous in identifying cell types that are recorded from (Barmack and Yakhnitsa, 2008; Brown et al., 2018) but this exceeded the scope of our experimental methods. Here, we used the cell’s location, its action potential as well as its firing pattern properties, especially the relation between the firing rate and its variability, as done previously (Vos et al., 1999). This approach has also been adapted and further perfected by others (Van Dijck et al., 2013). Finally, we did not fully monitor the animal’s postural, jaw, limb, or whisker movements. However, as we encouraged the animals to be immobile but attentive—the optimal behavior to observe stronger oscillatory periods—we also selected these periods for recording, noted sudden movement, and inspected the traces offline for artifacts. Future experiments should indeed address the posture/movement interface quantitatively, especially with the potential of information-rich differential phase-coding in sensorimotor planning and execution.

In conclusion, in the context of awake immobility, we have found that the 4–12 Hz GCL oscillations can help predict the spike timing of Golgi cells and Purkinje cell SSs. The LFPs represent a measure of synaptic activity influencing the GCL, potentially modulating large portions of the cerebellar cortex. Information could flow better across circuits, here through the cerebellar layers, using an oscillatory influence (Akam and Kullmann, 2010). Also, LFP oscillations could help in the coordination of spike timing even if cells are not rhythmic (Bush and Burgess, 2019), as we show here that a greater proportion of Golgi or Purkinje cell SSs are phase-locked than are outright rhythmic. Our study has focused on normal circuits, but oscillatory flow could also have implications in pathological circuits, influencing cerebellar and extra-cerebellar connectivity (Bares et al., 2010; Georgescu et al., 2018). As oscillations flowing through circuits can represent time (Buzsáki and Llinás, 2017), the understanding of oscillatory flow and the timing of unit activity through the cerebellar cortex, and outward, is of particular interest.

## Data Availability Statement

The datasets generated for this study are available on request to the corresponding author.

## Ethics Statement

The animal study was reviewed and approved by Concordia University Animal Research Ethics Committee.

## Author Contributions

ML and HG contributed equally to this work. ML, HG, CL, and RC designed and prepared the experiments. ML, HG, CS, CL, and RC acquired the data. ML, HG, JMPL, CL, and RC analyzed the data. ML, HG, CL, and RC wrote the manuscript, with all authors providing input. All authors contributed to the article and approved the submitted version.

## Conflict of Interest

The authors declare that the research was conducted in the absence of any commercial or financial relationships that could be construed as a potential conflict of interest.
