# Supplementary material for: Cerebellar Cortex 4–12 Hz Oscillations and Unit Phase Relation in the Awake Rat
Source: Front Syst Neurosci. 2020 Nov 10;14:475948. doi: 10.3389/fnsys.2020.475948 (PMC7683574; doi:10.3389/fnsys.2020.475948)
Supplement: Supplementary file 1 [file Data_Sheet_1.PDF]

Supplementary Material

To further document the effects of cerebellar LFP oscillations on LFP-LFP synchrony, we further documents the effect of oscillation on coherence in three rats (already in this paper), across full recording sessions. The database consists in 3 sessions from each of the 3 rats.

We separated the oscillatory periods from non-oscillatory ones using the algorithm described in the manuscript. The coherence in the alpha/theta frequency range was thus determined for the ensemble of oscillatory episodes, compared with periods when no oscillation was detected. A Kruskal-Wallis comparison showed ($p < 0.05$) an effect of oscillation content on alpha/theta frequency coherence :

Table 1. Effect of oscillatory detection on the LFP-LFP coherence.

3 rats * 3 sessions = 9 sessions	Periods	Coherence Mean	Coherence Median	25/75 prctiles
Non-oscillatory	23578	0.47	0.46	0.23/0.63
Oscillatory	5535	0.59	0.63	0.36/0.83

This provides a firmer basis of evidence for the role of oscillations in influencing LFP synchrony.
